# An Intermodal Correlation Study among Imaging, Histology, Procedural and Clinical Parameters in Cerebral Thrombi Retrieved from Anterior Circulation Ischemic Stroke Patients

**DOI:** 10.3390/jcm11195976

**Published:** 2022-10-10

**Authors:** Rebeka Viltužnik, Franci Bajd, Zoran Miloševič, Igor Kocijančič, Miran Jeromel, Andrej Fabjan, Eduard Kralj, Jernej Vidmar, Igor Serša

**Affiliations:** 1 Jožef Stefan Institute, 1000 Ljubljana, Slovenia; 2 Institute of Physiology, Faculty of Medicine, University of Ljubljana, 1000 Ljubljana, Slovenia; 3 Faculty of Mathematics and Physics, University of Ljubljana, 1000 Ljubljana, Slovenia; 4 Department of Diagnostic and Interventional Neuroradiology, Clinical Institute of Radiology, University Medical Centre, 1000 Ljubljana, Slovenia; 5 Department of Diagnostic and Interventional Radiology, General Hospital Slovenj Gradec, 2380 Slovenj Gradec, Slovenia; 6 Institute of Forensic Medicine, Faculty of Medicine, University of Ljubljana, 1000 Ljubljana, Slovenia

**Keywords:** ischemic stroke, cerebral thrombi, thrombectomy, computed tomography, multiparametric MRI, correlation analysis

## Abstract

The precise characterization of cerebral thrombi prior to an interventional procedure can ease the procedure and increase its success. This study investigates how well cerebral thrombi can be characterized by computed tomography (CT), magnetic resonance (MR) and histology, and how parameters obtained by these methods correlate with each other as well as with the interventional procedure and clinical parameters. Cerebral thrombi of 25 patients diagnosed by CT with acute ischemic stroke were acquired by mechanical thrombectomy and, subsequently, scanned by a high spatial-resolution 3D MRI including *T*_1_-weighted imaging, apparent diffusion coefficient (ADC), *T*_2_ mapping and then finally analyzed by histology. Parameter pairs with Pearson correlation coefficient more than 0.5 were further considered by explaining a possible cause for the correlation and its impact on the difficulty of the interventional procedure and the treatment outcome. Significant correlations were found between the variability of ADC and the duration of the mechanical recanalization, the deviation in average Hounsfield units (HU) and the number of passes with the thrombectomy device, length of the thrombus, its RBC content and many others. This study also demonstrates the clinical potentials of high spatial resolution multiparametric MRI in characterization of thrombi and its use for interventional procedure planning.

## 1. Introduction

Treatment of the cardiovascular diseases has been improved considerably over the last decades and the death rate due to these diseases has also declined; however, the burden to society remains high [1]. A significant contribution to this decline is in much better treatment of the large vessel occlusions, especially in acute ischemic stroke. Modern interventional treatment of ischemic stroke is based on the improved, more accurate, as well as faster, diagnostic imaging, either by computed tomography (CT) or magnetic resonance imaging (MRI) to confirm the diagnosis, which is immediately followed by vessel occlusion recanalization through biochemical thrombus degradation in thrombolysis [2] and/or by its mechanical removal in thrombectomy [3]. The origin of thrombi causing the occlusions may be in the heart, most often due to atrial fibrillation, or in atherosclerotic lesions within the affected vessel or proximal to it. However, it is not easy to distinguish between these two different thrombi etiologies, namely cardioembolic or arteriopathic, since they have practically identical histological characteristics [4]. In this analysis, MRI could be advantageous because it can produce images of various contrasts, which depend on different chemical and mechanical characteristics of thrombi. Thus, MRI is accurate in thrombi localization and can precisely determine thrombus structure and composition, which makes it an interesting in vivo alternative to conventional histology [5].

Platelets and red blood cells (RBCs) are two main cell components of thrombi. However, there is also a fibrin meshwork that acts as a bond with the cells and makes the thrombus structure rigid and resilient to recanalization procedures. The presence of blood cells in the thrombus is responsible for its porous structure and also defines its porosity [6]. A higher density of blood cells reduces the porosity and, therefore, makes the thrombus less permeable [7]. In addition, the fibrin meshwork can be densified, reducing the clot permeability in regions with an increased platelet concentration and also, in the case of establishing a fibrin meshwork cross-linking [8,9]. Regions with the denser fibrin meshwork have also an increased rigidity and are mechanically more stable [10]. Obviously, there is a link between the microscopic properties of thrombi and their susceptibility to recanalization, i.e., porosity of the thrombus structure defines its susceptibility to thrombolysis [11,12], while the rigidity of the fibrin meshwork determines the thrombus resistance to mechanical thrombectomy [13]. As the success of recanalization of the occluded artery in ischemic stroke critically depends on thrombus structure and composition, accurate characterization of these two properties prior to the recanalization procedure could significantly ease this interventional procedure and increase its success [14,15].

The most-often used imaging modality in the diagnosis of stroke is CT [16]. While native CT provides relatively poor contrasted images of patients suspected of stroke, its contrasted version used for CT angiography can clearly depict the site of the occluded artery and the extent of penumbra. Its rival, MRI, cannot compete with CT in terms of speed; however, it provides much better contrasted images. In MRI, the origin of image contrast is quite different than in CT. It is primarily based on NMR relaxation times that depend on the molecular mobility, chemical environment, local magnetic centers, etc., [17]. In addition, the image contrast can be made sensitive to diffusion, perfusion and also to the environmental factors, i.e., temperature, pH, etc., [18]. In CT, contrast is based on X-ray attenuation of tissues in the body. This increases with higher atomic number tissues and, therefore, denser tissues [19]. In the case of MRI, a very efficient method in diagnosis of ischemic stroke is the diffusion-weighted imaging (DWI) or its quantitative version, the apparent diffusion coefficient (ADC) mapping [20]. This detection of ischemic stroke is based on slow water diffusion in the ischemic regions of the brain due to an increased intra- to extra-cellular water ratio [21]. The affected regions of the brain appear brighter in DWI and exhibit lower ADC values than normal tissue. The DWI imaging sequence utilizes a pair of strong bipolar magnetic field gradients that result in additional signal attenuation by a factor exp(-*bD*) where *D* is the apparent diffusion coefficient and *b* is the instrumental diffusion-weighting factor dependent on the bipolar gradient pulse. Ischemic stroke was also investigated by *T*_2_* and *T*_2_ mapping, where it was found that *T*_2_* is sensitive on the oxygenation state of the affected brain region [22], while *T*_2_ correlates with the time from the stroke onset [23]. Both MRI methods, namely *T*_2_ and ADC mapping, have been used previously for the characterization of blood clots, e.g., to study changes in the structure and composition of artificial blood clots during their dissolution [24] and extracted pulmonary thromboemboli [25].

This study is a continuation of our previous studies on cerebral thrombi retrieved by mechanical thrombectomy from patients diagnosed with acute ischemic stroke. In these studies, the thrombi were evaluated in vitro after their thrombectomy by multiparametric MRI [26] or assessed in vivo by CT [27] and correlation was made with either of these techniques, and the results of histology, clinical and the interventional procedure data was tested. Herein, all the available data on the thrombi (MRI, CT, histology, interventional procedure and clinical data) are evaluated as a whole, providing opportunity for a comprehensive correlation study of these parameters characterizing thrombi.

## 2. Materials and Methods

### 2.1. Patient Selection and Study Design

The study was designed in a way to minimally interfere with the normal clinical interventional procedure for the management of patients with the suspicion of acute stroke. Upon admission to the Neurology Clinic of University Medical Center Ljubljana, due to neurological symptoms suggesting brain stroke, these patients underwent standard diagnostic procedure that included urgent clinical examination followed by an urgent multimodal CT scan. Patients diagnosed with acute ischemic stroke received a standard full dose of the rt-PA (0.9 mg/kg, maximum 90 mg, 10% in bolus first and then the remaining 90% intravenously in 1 h) systemic thrombolytic treatment within 4.5 h of onset. If the clinical stroke signs persisted after the thrombolytic treatment, the patients received further therapy by mechanical thrombectomy at an average of 130 min after initiation of thrombolysis. This was undertaken by the standard mechanical recanalization procedure performed by the skilled interventional neuroradiologist using the thrombectomy device (Trevo^®^stent retriever, 4 × 20 mm, Stryker Neurovascular, Kalamazoo, MI, USA).

**Figure 1:**
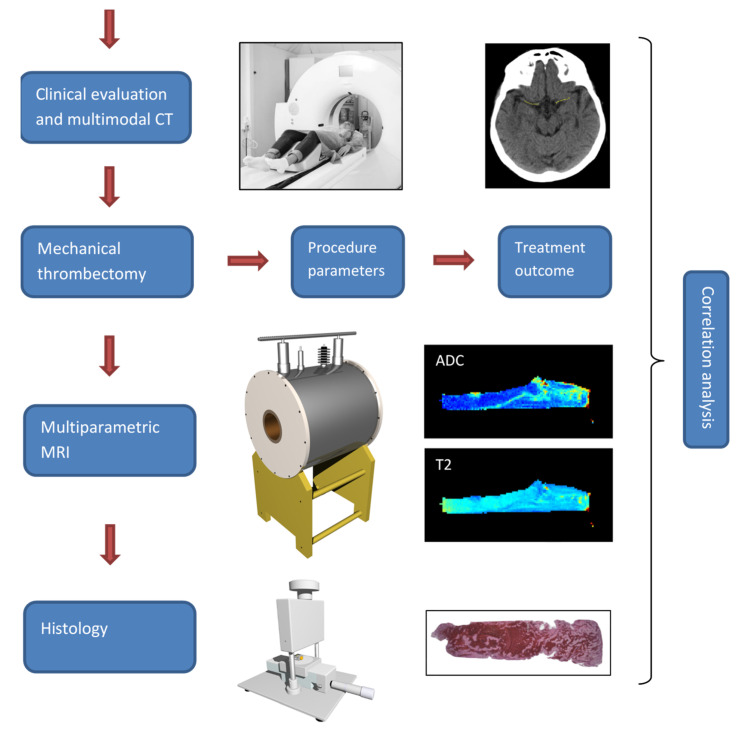
Study design. Patients suspected with an acute stroke underwent clinical examination and a CT scan. If ischemic stroke was confirmed and the thrombolysis was unsuccessful, the patients received further therapy by mechanical thrombectomy. The thrombi retrieved from the MCA vessel segment were further analyzed by high spatial resolution multiparametric MRI and histology. Different parameters obtained from imaging (CT, MRI and histology) and the interventional procedure and treatment were then analyzed for possible correlations.

All the relevant clinical and procedure parameters, e.g., age, time to thrombolysis, duration of mechanical recanalization, number of passes with the thrombectomy device, were recorded for each patient. The thrombi, retrieved from the M1 segment of the middle cerebral artery (MCA), of *n* = 25 patients (mean age = 73 ± 11 years, 16 males and 9 females) were preserved and additionally examined by high spatial resolution multiparametric MRI and immunohistochemistry. Prior to the MRI, the samples were rinsed with isotonic saline of 0.9% *w*/*v* of NaCl, pH 7.4 and closed in Teflon tubes to prevent tissue desiccation during the MR scanning, which was performed within 24 h after the retrieval of the thrombus. Thereafter, samples were processed for histological analysis. All the three imaging modalities (CT, MRI and histology), as well as the interventional procedure and treatment outcome, were the source of several different parameters that were then analyzed pairwise for possible correlations. The design of this study is shown in Figure 1.

This study was approved by the Ethical Committee of the National Ministry of Health of the Republic of Slovenia, approval No. 0120-99/2021/7 from 21 May 2021. The study was performed in agreement with the informed-consent policy.

### 2.2. CT Imaging

The CT examination of patients suspected with stroke included non-contrast enhanced (NCE) sequential CT scans and contrast CT angiography (CTA) scans. The NCE CT scan was a two-part scan: scull-base and cerebral. Both scans were sequential and had the following parameters for the scull base region: 120 kV, 265 mAs, matrix 1024 × 1024, slice thickness 3 mm, collimation 20 × 0.6, rotation time 1 s, window width 90–190, window center 38, and for the cerebral region: 120 kV, 310 mAs, matrix 1024 × 1024, slice thickness 4.8 mm, collimation 24 × 1.2, rotation time 1 s, window width 80, window center 38. CT scanning was performed on a Siemens Sensation Open 40 CT scanner (Siemens, Erlangen, Germany) at the Neurology Clinic of University Medical Centre Ljubljana.

### 2.3. Multi-Parametric MRI

The MR imaging was performed on an experimental MRI scanner consisting of a 2.35 T (100 MHz proton frequency) horizontal bore superconducting magnet (Oxford Instruments, Abingdon, UK) equipped with a Bruker micro-imaging system (Bruker, Ettlingen, Germany) for MR microscopy with a maximum imaging gradient of 300 mT/m and an Apollo spectrometer (Tecmag, Houston, TX, USA). Each thrombus sample was inserted into a 10 mm micro-imaging probe and then analyzed by a multi-parametric MRI protocol, which consisted of the following 3D imaging sequences: *T*_1_-weighted, ADC mapping and *T*_2_ mapping. The corresponding imaging sequences were based on the spin-echo, pulsed gradient spin-echo (PGSE) [28] and Carr–Purcell–Meiboom–Gill (CPMG) multi-echo [29] sequences, respectively. MR imaging was performed with the field of view of 20 × 10 × 10 mm^3^ and the imaging matrix equal to 128 × 64 × 64 (*T*_1_-weighted) or to 128 × 64 × 16 (mapping) so that the resolution was equal to 156 μm isotropic (*T*_1_-weighted) or 156 μm in-plane (mapping). Other sequence specific parameters were equal to: TE/TR = 5/100 ms, signal averages 10 for the 3D spin-echo sequence, TE/TR = 34/1035 ms, *b* = 0, 260, 620, 1250 s/mm^2^, signal averages 2 for the 3D PGSE sequence and iTE/TR = 16/1930 ms, number of echoes 8, signal averages 2 for the 3D multi-echo sequence. MRI scanning of thrombi was performed at a constant room temperature of 22 °C.

### 2.4. Histology

For histological analysis, which followed the MRI examination, the thrombi were fixed in 4% buffered formaldehyde for 48 h, cut longitudinally and, if necessary, transversely, processed on the Tissue processor Thermo Scientific Excelsior ES and embedded in paraffin. Finally, serial 5-μm-thick sections were cut from each sample and stained by hematoxylin-eosin (HE) for the determination of a general thrombi composition, by Monoclonal Mouse Anti-Human CD235a Glycophorin A, DakoCytomation, Denmark (GPA) for RBC content determination and by Monoclonal Mouse Anti-Human CD61, DakoCytomation, Denmark (CD61) for platelet content determination. The immunohistochemical (IHC) stainings were performed using a Ventana BenchMark automatic stainer with a streptavidin-biotin peroxidase complex and diaminobenzidine tetrahydrochloride as a chromogen. Stained sections of the thrombi were examined by a Nikon Eclipse E600 optical microscope (Nikon, Düsseldorf, Germany) equipped with Nikon Plan Fluor objectives and with a high-resolution CCD camera. The system was controlled by the Nikon NIS Elements software allowing precise microphotography of the samples with optimal image contrast.

### 2.5. Processing of Images

CT images were processed by the ImageJ program (NIH, Bethesda, MD, USA) in order to extract information on the thrombus location, its length (L) and especially its radio-density measured in the units of Hounsfield (HU). The first two parameters, i.e., location and length were determined from CTA images, while the HU values were measured from NCE CT images. In order to obtain more accurate HU values, images of three consecutive slices central to the thrombus were stacked and the HU value profile along the line of the central part of the thrombus was measured. Special care was taken for the line not to include the HU values from the vessel wall or possible calcifications next to the thrombus. The same operation was repeated on the non-occluded symmetrically located MCA artery to obtain a reference HU value profile. Both profiles, from the occluded and non-occluded site, were then analyzed further to determine the average HU value (HU_avg) of the profile and its standard deviation (HU_var). Finally, differences of these two parameters between the occluded and non-occluded MCA site were calculated, thus obtaining ΔHU_avg and ΔHU_var parameters.

MR and histological images were processed by using in-house written image-analysis software that was developed within the Matlab programming environment (MathWorks, Inc., Natick, MA, USA). In the MR image processing software, ADC and *T*_2_ values were calculated pixelwise from the masked DWI and multi-spin-echo images of all slices through the entire thrombi volumes by fitting the data to the exponentially decaying function. Calculated ADC and *T*_2_ maps were analyzed for average and within-sample variation of their values in the thrombus region (in all slices) thus obtaining four parameters: ADC_avg, ADC_var, T2_avg and T2_var. The within-sample coefficient of variation can be considered as a measure for heterogeneity of the thrombus.

Red blood cell (RBC) proportions of each thrombus was determined from its standard (hematoxylin-eosin stained) histological image of the central slice across the thrombus. This analysis included the following steps: correction of uneven illumination [30], calculation of the image histogram to determine optimal threshold for the discrimination between RBC-rich and platelet-rich part of the thrombus and, finally, the calculation of the RBC proportion as the ratio between the area of the thresholded RBC region and the total thrombus area.

### 2.6. Clinical and Intervention Procedure Parameters

On admission to the urgent medical-care center and at discharge from the hospital, the neurological status of each patient was graded according to the modified Ranking Scale (mRS) for the stroke and also to the NIH stroke scale (NIHSS). This enabled following of four clinical parameters: imRS, ΔmRS, iNIHSS and ΔNIHSS, where character i denotes initial (grade on admission) and Δ difference between final grades at discharge and initial grades on admission.

In addition to the clinical parameters of each patient, the corresponding interventional procedure parameters were also recorded. These parameters include the time to thrombolysis (tt_Lysis), duration of mechanical recanalization (t_MeR) and number of passes with the thrombectomy device (# Passes). t_MeR implies successful recanalization of the occluded vessel and it was determined as the time from groin puncture to recanalization through the occluded artery by complete mechanical removal of the occluding thrombus.

### 2.7. Statistical Analysis

Parameters obtained in the study: MRI (ADC_avg, ADC_var, T2_avg, T2_var), CT (ΔHU_avg, ΔHU_var, L), histology (RBC %), procedure (tt_Lysis, t_MeR, # Passes) and clinical (imRS, ΔmRS, iNIHSS, ΔNIHSS) were tested for the possible correlations. Specifically, the Pearson and Spearman correlation coefficients were calculated for each possible pair of the parameters using Excel statistical tools (Microsoft, Seattle, WA, USA). The correlations were considered important when the absolute value of the correlation coefficient was higher than 0.5 and indicative when it was higher than 0.3.

## 3. Results

**Figure 2:**
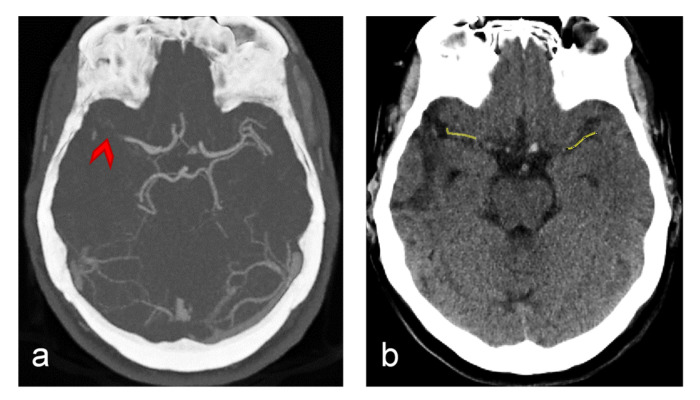
Representative brain CT images of an ischemic stroke patient in a slice with the middle cerebral (MCA) artery: (**a**) CT angiograph (CTA) image and (**b**) non-contrasted CT (NCT) image of the same slice. Red arrow in the CTA image points to the occlusion (thrombus), while yellow lines in the NCT image correspond to the lines along which HU values were measured in the occluded site (left) and in the symmetrical positioned non-occluded site (right).

CT brain imaging is the most common imaging modality in stroke diagnosis. In Figure 2 are shown two such images, one contrasted (CTA, a) and one non-contrasted (NCT, b). Both images are of the same slice positioned along both middle cerebral arteries (MCAs). In the CTA image, all the major blood vessels as well as the location of the occluded MCA to where points a red arrow are clearly seen. The NCT image is displayed with a different HU brightness window that is adjusted for displaying of soft tissues. Along the occluded MCA vessel, a yellow line is drawn and then another such line is drawn in the symmetric nonoccluded MCA site. Along these lines, HU values were measured and then processed to obtain ΔHU_avg and ΔHU_var parameters.

**Figure 3:**
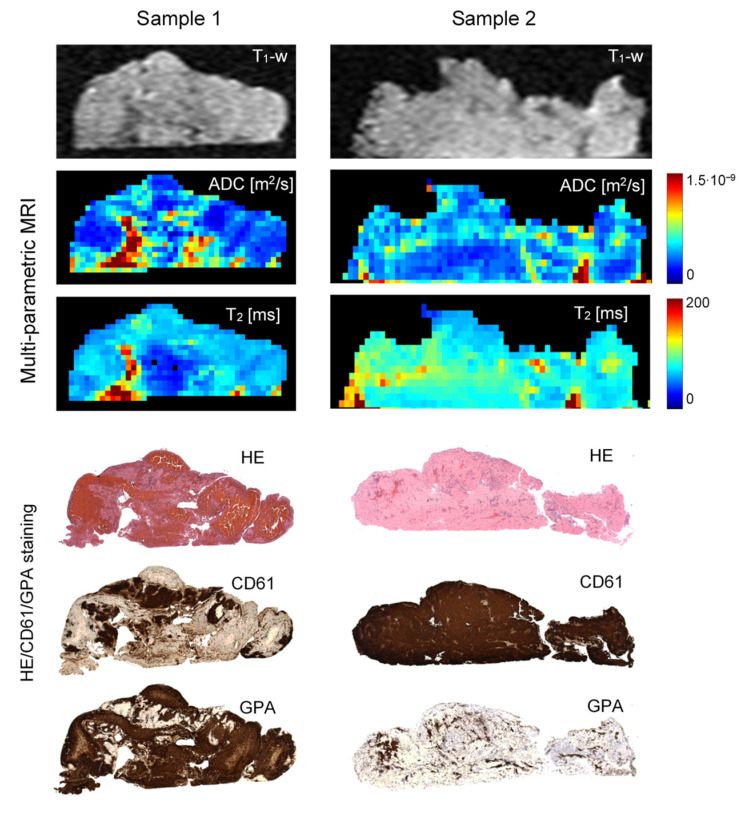
Two representative cerebral thrombi of a different structure are presented by central-slice *T*_1_-weighetd images, ADC and *T*_2_ maps along with the corresponding histological (HE/CD61/GPA) images. Lower ADC and *T*_2_ values, shown in dark blue, in maps of Sample 1 correspond to compact RBC-rich regions, while lower ADC and *T*_2_ values in Sample 2 correspond to compact platelet-fibrin-rich regions. CD61 staining is specific to platelets (dark brown), while GPA staining is specific to RBC components (dark brown).

Figure 3 depicts *T*_1_-weighted images and ADC and *T*_2_ maps along with the corresponding histological background-corrected HE/GPA/CD61 images of the two representative cerebral thrombi of a different structure and composition. It can be seen that both samples show quite significant variability of ADC values across the thrombi, while their *T*_2_ values are somewhat more uniform. In general, discrimination among different regions of low water mobility is better in ADC maps than in the corresponding *T*_2_ maps. From the comparison between ADC/*T*_2_ maps and IHC images, it can be seen in Sample 1 that regions with low ADC/*T*_2_ values correspond to the regions abundant with RBCs. This reduction of ADC/*T*_2_ values can be explained by the reduced mobility of water molecules due to a high RBC compaction resulting in a significant reduction of the extracellular space. Another mechanism of the ADC/*T*_2_ reduction can be seen in Sample 2, where high platelet concentration locally affects fibrin fiber architecture, resulting in a fibrin meshwork contraction and, therefore, a pore size reduction, and also in an extracellular serum expulsion [9]. The regions with intermediate ADC/*T*_2_ values are mainly composed of a mixture of platelet-rich and RBC-scarce regions. In addition, the central part of Sample 1 also consists of a few smaller inclusions with higher ADC/*T*_2_ values. The inclusions correspond to regions of entrapped serum that can be clearly identified in the underlying.

Histological images as tissue voids. Differences between both samples are also well observed in histological and IHC images. Specifically, in CD61 images a positive reaction to the platelet content, and in GPA images a positive reaction to RBCs are shown in brown. These two thrombi samples were also significantly different in RBC proportions, 63 ± 7% (Sample 1) vs. 5 ± 3% (Sample 2), as determined from the corresponding histological/IHC images.

**Figure 4:**
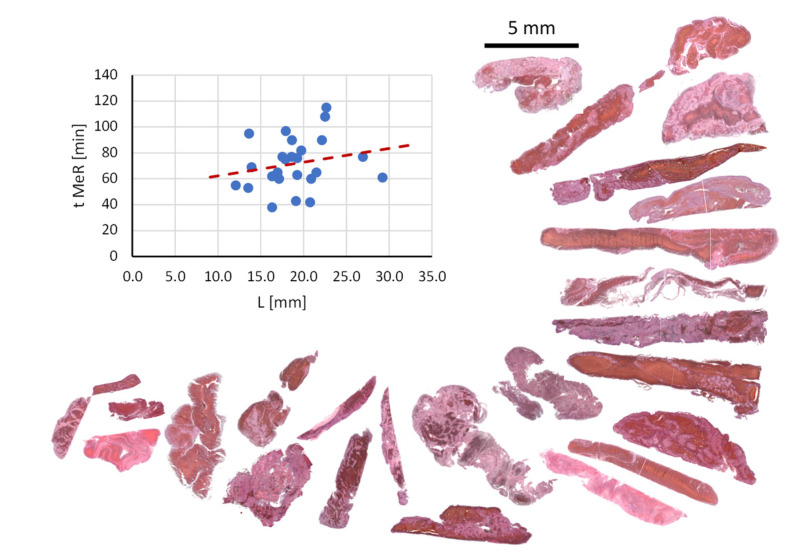
A correlation plot between the mechanical thrombectomy procedure time and the thrombus length. From the plot it can be seen that the procedure time increases with the thrombus length. The figure also shows histological images of all the thrombi in the study ordered by their lengths.

A relation between the duration of mechanical thrombectomy procedure (t_MeR) and the thrombus length (L) as measured in vivo from CT images prior to the thrombus retrieval is shown by a graph in Figure 4. The figure also shows histological images of the thrombi ordered lengthwise. The result supports existing findings of other groups, namely that the duration of mechanical thrombectomy procedure increases with the thrombus length [31]. However, it can also be seen that this general trend has also some isolated exceptions where shorter thrombi have been hard to retrieve or longer thrombi were easy to retrieve. This indicates that, in addition to the thrombus length, its composition also plays a very important role in the mechanical thrombectomy procedure. Large diversity of thrombi composition can be seen well in the presented histological images. The histological analysis of the thrombi also shows that the longer thrombi have in average more complex composition, namely, they often contain several inclusions of compact RBC-rich areas surrounded by thin platelet-fibrin layers.

All data of the thrombi qualified for the study are presented in Table 1. The data are grouped according to the categories into MRI, CT, histology, interventional procedure and clinical parameters; all together there are 16 parameters on each thrombus. This data then enabled the calculation of correlations between different pairs of parameters. The calculated correlation coefficients are displayed in Table 2 with Pearson correlation coefficients above the diagonal cells and Spearman correlation coefficient below the diagonal cells. The correlation pair of a cell is identified by the parameters corresponding to the intersection of the row and column of the cell. Cells with correlation coefficients considered significant are highlighted in green (0.5 ≤ |ρ| < 1), the semi significant correlation coefficients are highlighted in yellow (0.3 ≤ |ρ| < 0.5), while those that are considered insignificant are left unmarked (|ρ| < 0.3).

**Figure 5:**
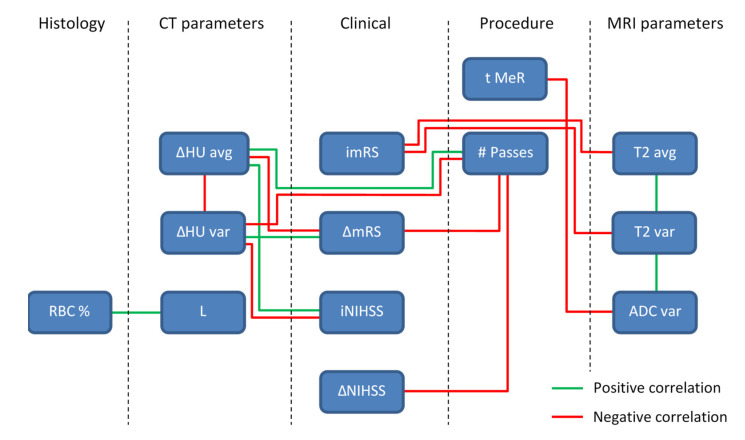
A diagram of significant correlations among different thrombi parameters. The parameters are organized according to their category in histology, CT, clinical, procedure and MRI parameter groups and existing correlations between parameter pairs are plotted with a green line for positive correlations and a red line for negative correlations.

For easier interpretation of the results in Table 2, all significant correlations according to the Pearson correlation coefficient are presented by a diagram in Figure 5. In the diagram, all the parameters with significant correlations are organized by their category and interconnected with a green line, for a significant positive correlation, or a red line, for a significant negative correlation. From this diagram, can also be seen that some of the parameters have quite substantial number of significant correlations with other parameters, e.g., ΔHU_avg, ΔHU_var and # Passes, while others are more isolated, e.g., L and RBC %. It can also be seen that the negative correlations dominate over positive correlations.

**Figure 6:**
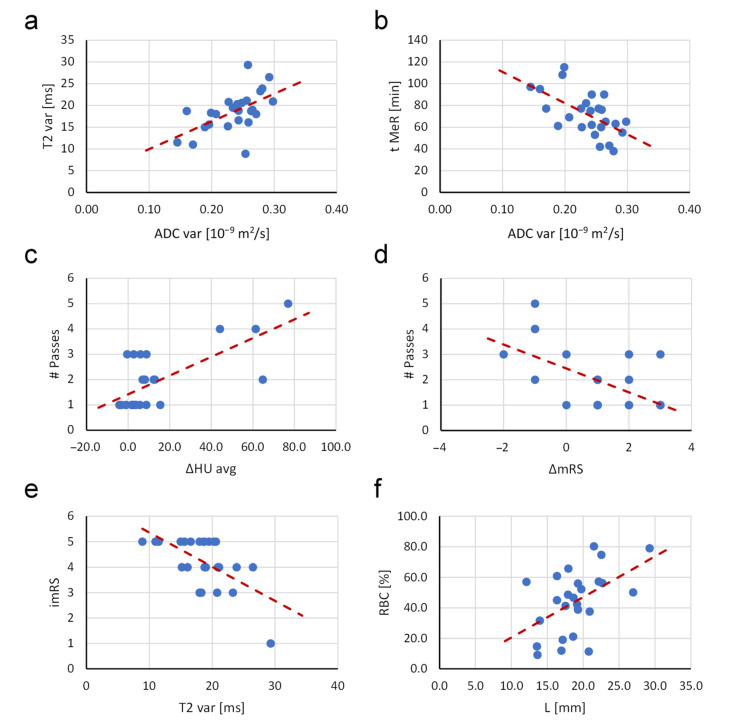
Plots of correlations between selected pairs of thrombi parameters: (**a**) T2_var vs. ADC_var, (**b**) t_MeR vs. ADC_var, (**c**) # Passes vs. ΔHU_avg, (**d**) # Passes vs. ΔmRS, (**e**) imRS vs. T2_var, (**f**) RBC % vs. L. Red dashed lines indicate trend lines of the data.

Some of the more important and characteristic correlations are shown in Figure 6. The variabilities of both MRI parameters i.e., *T*_2_ and ADC, correlate positively and so do the number of passes with the thrombectomy device and the difference in HU between the occluded and symmetric normal site, as well as the proportion of RBCs and the thrombus length. The remaining of the presented correlations in Figure 6 are negative. Such correlations are found between the duration of mechanical recanalization and the variability of ADC, the number of passes with the thrombectomy device and the difference between the final and initial modified Rankin score, the initial modified Rankin score and the variability of *T*_2_. Red dashed lines in the plots indicate the trend lines.

## 4. Discussion

In this study, the retrieved cerebral thrombi were characterized by means of native (non-contrasted) CT, multi-parametric MRI and histology. In addition, the characterization by these imaging modalities was complemented with clinical and interventional procedure data and, thus, enabled a vast selection of parameters on each thrombus. Specifically, 16 parameters were measured: four MRI, three CT, one histological, three interventional procedure and five clinical. This enabled a comprehensive investigation of relations among these parameters by two standard correlation tests, Pearson and Spearman. Correlation analyses were possible since the thrombi were characterized quantitatively by the imaging methods of which contrast depends exclusively on the intrinsic tissue properties. Two such methods, that were used in this study are ADC and *T*_2_ mapping that produce images of water mobility and *T*_2_ NMR relaxation time. In several existing studies involving MRI, cerebral thrombi were scanned by the non-quantitative methods, e.g., *T*_2_-weighetd, FLAIR and GRE [32] or *T*_2_*-weighted GRE and SWI [5]. These methods produce images in which contrast, in addition to the tissue properties, also depends on the scanning parameters, and so would the obtained correlation coefficients. The relations among some of the parameters analyzed in this study were also known from some previous studies; however, these studies were focused to a more limited set of parameters or were performed on other type of blood clots, e.g., relation between thrombus length and the number of stent retrievals [31], relation between RBC content and *T*_1_ and *T*_2_ NMR relaxation times in artificial blood clots [33].

Table 2 clearly shows which parameter correlations among these are significant (highlighted in green) or indicative (highlighted in yellow). For the most part, these results meet expectations, e.g., more compact thrombi have lower *T*_2_, ADC and higher ΔHU average values and also higher variability of these parameters due to their often-heterogeneous structure. Such values of ADC and *T*_2_ of the compact thrombi can be explained by an increased tortuosity and decreased porosity that is responsible for ADC decrease [34], while an increase in surface-to-volume ratio in these thrombi causes an increased surface-induced relaxation; therefore, *T*_2_ decreases [35]. The origin of surface-induced relaxation can also be calcifications in thrombi [36]. However, as can be seen from the histological sections in Figure 4, thrombi included in this study did not contain any noticeable (macroscopic) calcifications. Compacted thrombi also have higher radio-density (HU values) mainly due to the higher iron concentration in closely packed RBCs [37]. Since compact thrombi lyse slower and pose greater challenge for mechanical recanalization, all these parameters are also associated with longer duration of mechanical recanalization (t_MeR), higher number of passes with thrombectomy device (# Passes) and longer times to thrombolysis (tt_Lysis), which is also supported by the results in Table 1 and Table 2. In addition, from these results, it can be seen that the thrombi with such MRI and CT parameters have higher initial NIH stroke scale and modified Rankin scale values (iNIHSS and imRS) and poorer decrease in these values after the treatment (ΔNIHSS and ΔmRS). Among the parameters that can already be obtained in vivo is also the thrombus length (L). This parameter correlates positively with t_MeR (Figure 4) and interestingly also with RBC % (Figure 6f). The impact of thrombus histological characteristics (structure, composition, RBC proportion and length) on the performance of the thrombectomy device was discussed already in [14] where the link between the histological characteristics, the mechanical properties and therefore mechanical recanalization was exposed. Thrombus length is not only important for the success of mechanical thrombectomy, but also has a great impact on thrombolysis [38]. In our study, this link cannot be seen because thrombolysis was unsuccessful and was in all cases continued with mechanical thrombectomy as soon as it was assessed that the progress of thrombolysis is poor.

The correlations discussed above, and in Section 3, refer to the Pearson correlation coefficients, i.e., above diagonal elements in Table 2. Below the diagonal elements of this table are Spearman (rank) correlations coefficients. From the results in this table, it can be seen that in most cases the Pearson and Spearman correlation tests yield the same pairs of parameters with significant correlations, and only the significance of the correlations differs between the two.

An interesting addition to our ADC and *T*_2_ mapping MRI techniques for characterization of thrombus composition would be magnetization transfer (MT) imaging [39]. In MT, selective off-resonance radio-frequency irradiation of the protein-bound protons enables the detection of the protein-rich fibrin-to-platelet regions. MT enables the quantitative determination of the individual protein components in thrombi. This is an entirely different characterization method than ADC or *T*_2_ mapping, which are sensitive to changes in water diffusion in thrombus porous structure or to microstructure-induced changes in *T*_2_ NMR relaxation time. Recently, the MT technique was efficiently employed for the characterization of the human arterial thrombi ex vivo [40].

This study was performed on the M1 segment of the MCA for two reasons. The first is that this segment can be optimally visible on CT images and the second is that thrombi can be best retrieved from this site, i.e., mostly without their disintegration. This was important for the success of the study, namely, the second part the study was designed for in vitro investigation of thrombi structure and composition by histology and by MR microscopy, which could not be undertaken on the disintegrated thrombi.

Perhaps, the biggest limitation of this study is the relatively low number of patients involved in the study (*n* = 25). The small sample group could result in a reduced accuracy of the calculated correlation coefficients [41]. Correlation bias is higher with parameter pairs with lower correlation coefficients. From this perspective, the results of this study can be considered as preliminary results. An interesting expansion of the sample group would be the inclusion of patients who were treated only by mechanical thrombectomy. This would allow comparison of the measured parameters between our existing group (thrombolysis followed by thrombectomy) with this group (thrombectomy only). However, the narrow selection criteria would have made this group even smaller, so no such comparison can be made with the available data. Another limitation of this study is its two-way design that includes in the first stage in vivo experiments (CT) and in the second stage in vitro experiments (MRI, histology). In case of clinical application of the results of this study, all data can only be acquired in vivo.

As can be seen from Table 1, the selection of patients for the study was such that all range of stroke severity was covered (see initial NIHSS and mRS grades). Thus, the study group includes also two patients with most severe symptoms (iNIHSS = 42) and two with very mild symptoms (iNIHSS < 6) where mechanical thrombectomy is usually not performed due to lack of validation; however, it is not contraindicated. In our opinion, such selection of the study group enables reaching extremes of the measured parameters and, thus, contributes to accuracy of the studied correlations among the parameters.

The prediction of the recanalization procedure duration and optimization of the means for achieving the recanalization could help to improve the planning of interventions. This would include optimal selection among thrombolysis alone, in combination with thrombectomy or thrombectomy alone, finer thrombolytic treatment adjustment and optimization in selection of the thrombectomy device. Based on the methods used and the results of this study, predictive models for the optimal intervention procedure can be designed. However, the use of such a model in the clinical environment would mainly be hampered by limitations in clinical diagnostic imaging. Current technology of MRI does not enable clinical imaging with microscopic resolution, especially not in acceptable time. Therefore, there is currently no efficient substitute for the standard histology. However, there is constant improvement in diagnostic imaging technology, not only in MRI, but also in CT, especially in soft tissue contrast. Therefore, the future prospects for possible optimization of the interventional procedure based on the accurate characterization of the thrombus before the intervention with diagnostic imaging are good.

## 5. Conclusions

Multi-modality imaging of the cerebral thrombi, by CT, MRI and histology, enables the precise assessment of their intrinsic properties, such as composition, structure and compactness. Furthermore, it was shown that various parameters obtained by imaging, e.g., variability of *T*_2_ and ADC, difference in HU between occluded and normal site, correlate well with some of the interventional procedure parameters, e.g., number of passes with the thrombectomy device, duration of the mechanical recanalization. The study was designed in vitro; however, its results have also a clinical relevance as they can help better planning of the interventional procedures in the patients diagnosed with ischemic stroke.

## Figures and Tables

**Table 1:** MRI, CT, histological, interventional procedure and clinical parameters of patients diagnosed with ischemic stroke due to occlusion in the MCA artery that were qualified for the study.

	MR Parameters				CT Parameters			Histol.	Procedure Parameters			Clinical Parameters					
Pt. #	ADC Avg [10^−9^ m^2^/s]	ADC Var [10^−9^ m^2^/s]	*T*_2_ Avg [ms]	*T*_2_ Var [ms]	ΔHU Avg	ΔHU Var	L [mm]	RBC % [%]	Tt Lysis [min]	t MeR [min]	# Passes	Age [yrs]	Tx before Stroke	iNIH SS	ΔNIH SS	imRS	ΔmRS
1	0.50	0.26	89	21	−3.3	−0.22	20.8	11.5	110	42	1	77	/	21	20	4	3
2	0.48	0.24	90	17	12.8	−4.40	16.3	45.1	240	62	2	77	/	17	9	5	1
3	0.72	0.30	79	21	3.4	0.80	17.0	12.0	120	65	1	63	/	23	16	4	1
4	0.64	0.25	59	9	2.5	7.08	17.5	41.4	95	77	1	83	AA	18	15	5	3
5	0.38	0.15	78	12	−0.5	−0.31	17.9	65.9	145	97	3	43	/	26	20	5	2
6	0.61	0.28	85	23	5.9	1.95	16.3	61.0	89	38	3	78	/	7	7	3	3
7	0.57	0.24	73	19	−4.2	−0.49	22.2	57.3	50	90	1	58	/	13	10	4	3
8	0.73	0.23	94	15	−1.1	−1.63	26.9	50.3	107	77	1	77	/	14	11	4	1
9	0.48	0.20	85	18	8.1	4.60	22.7	56.3	110	115	2	85	ACAA	5	3	3	1
10	0.61	0.23	64	20	44.1	−24.60	19.7	52.4	165	82	4	81	/	26	−16	5	−1
11	0.55	0.23	83	21	−1.1	1.83	17.1	19.1	148	60	1	66	/	12	12	3	3
12	0.52	0.21	76	18	1.7	−1.83	13.9	31.8	185	69	1	72	AA	6	3	3	2
13	0.44	0.20	73	16	12.3	−0.46	22.5	74.7	125	108	2	62	/	19	7	5	1
14	0.69	0.28	101	24	76.9	−53.00	19.3	38.9	81	63	5	79	/	18	−2	4	−1
15	0.50	0.24	85	20	64.7	−44.40	17.9	48.8	120	75	2	91	/	42	0	5	−1
16	0.50	0.17	73	11	1.7	−1.30	18.6	21.3	140	77	1	73	/	15	11	5	1
17	0.71	0.25	88	21	1.8	−0.63	13.5	14.8	67	53	1	72	AA	14	12	5	2
18	0.71	0.27	73	18	15.4	0.87	19.1	42.4	90	43	1	85	AC	22	19	5	1
19	0.56	0.26	109	29	3.8	1.18	20.9	37.7	60	60	1	73	/	3	2	1	1
20	0.62	0.26	77	16	8.8	0.56	19.3	56.0	120	76	3	86	AC	11	−29	4	−2
21	0.36	0.19	93	15	8.7	−1.99	29.2	79.1	90	61	1	79	/	19	9	5	0
22	0.37	0.16	71	19	2.6	0.24	13.6	9.3	110	95	3	70	/	16	7	5	0
23	0.53	0.27	84	19	6.9	0.67	21.5	80.4	25	65	2	65	/	16	13	4	2
24	0.65	0.29	89	27	5.6	−1.20	12.1	57.1	75	55	1	53	/	13	10	4	3
25	0.63	0.26	80	19	61.3	−42.60	18.7	46.8	105	90	4	69	AA	42	0	5	−1

#—index/number; ADC—Apparent diffusion coefficient, *T*_2_—Transversal NMR relaxation time, HU—Hounsfield units, avg—Sample mean value, var—Within sample variability (standard deviation), RBC %—Percentage of red blood cells in the thrombus, /—None, AA—Anti-aggregation, AC—Anticoagulant, NIHSS—NIH stroke scale, mRS—Modified Rankin scale, i—Initial, Δ—Difference between final and initial.

**Table 2:** Correlation coefficients between different pairs of thrombi parameters. A correlation pair is defined by parameters of row and column of the corresponding cell. Above the table diagonal cells are shown Pearson correlation coefficients, while below them are shown Spearman correlation coefficients. Cells with the absolute value of the correlation coefficient higher than 0.5 are highlighted in green, while those between 0.3 and 0.5 are highlighted in yellow. To better distinguish between Pearson and Spearman correlation coefficients, the diagonal elements are written in red.

	Pearson	ADC Avg	ADC Var	*T*_2_ Avg	*T*_2_ Var	ΔHU Avg	ΔHU Var	L	RBC %	Tt Lysis	t MeR	# Passes	Age	iNIHSS	ΔNIHSS	imRS	ΔmRS
Spearman																	
ADC avg		1.00	0.77	0.05	0.29	0.18	−0.16	−0.16	−0.22	−0.28	−0.40	−0.02	0.18	−0.01	−0.09	−0.12	0.02
ADC var		0.71	1.00	0.24	0.57	0.26	−0.21	−0.18	−0.06	−0.35	−0.59	0.01	0.19	0.04	−0.10	−0.26	0.06
*T*_2_ avg		0.06	0.24	1.00	0.60	0.12	−0.18	0.23	0.03	−0.23	−0.37	−0.04	0.04	−0.22	0.04	−0.52	−0.05
*T*_2_ var		0.33	0.62	0.43	1.00	0.21	−0.22	−0.24	−0.16	−0.33	−0.43	0.08	−0.03	−0.20	−0.14	−0.61	0.03
ΔHU avg		0.01	0.30	0.08	0.10	1.00	−0.97	0.00	0.12	0.04	0.09	0.70	0.35	0.64	−0.44	0.25	−0.68
ΔHU var		0.05	0.14	−0.24	0.05	−0.35	1.00	0.02	−0.03	−0.04	−0.07	−0.66	−0.24	−0.66	0.38	−0.23	0.62
L		−0.15	−0.19	0.14	−0.25	0.08	−0.01	1.00	0.50	−0.23	0.21	−0.08	0.21	0.01	−0.03	−0.03	−0.21
RBC %		−0.22	−0.06	0.04	−0.25	0.28	−0.06	0.47	1.00	−0.20	0.25	0.20	−0.10	0.04	−0.19	0.08	−0.10
tt Lysis		−0.38	−0.50	−0.30	−0.38	0.01	−0.19	−0.19	−0.21	1.00	0.15	0.10	0.07	0.12	−0.15	0.21	−0.17
t MeR		−0.32	−0.58	−0.48	−0.54	0.01	−0.13	0.31	0.24	0.34	1.00	0.27	−0.15	0.15	−0.23	0.21	−0.30
# Passes		−0.18	0.01	−0.09	0.04	0.60	−0.26	0.02	0.29	0.17	0.38	1.00	0.16	0.34	−0.55	0.19	−0.60
Age		0.08	0.06	0.07	−0.13	0.46	−0.02	0.21	−0.05	0.10	−0.19	0.09	1.00	0.04	−0.42	0.01	−0.45
iNIHSS		−0.04	0.00	−0.19	−0.17	0.37	−0.42	0.05	0.02	0.22	0.17	0.28	0.03	1.00	0.01	0.65	−0.43
ΔNIHSS		0.08	0.01	−0.09	−0.16	−0.60	0.40	−0.12	−0.15	−0.10	−0.26	−0.57	−0.37	0.17	1.00	0.11	0.72
imRS		−0.19	−0.31	−0.36	−0.52	0.30	−0.41	−0.09	0.04	0.20	0.28	0.19	0.07	0.73	0.12	1.00	−0.27
ΔmRS		0.06	0.10	−0.03	0.15	−0.70	0.49	−0.26	−0.01	−0.22	−0.34	−0.54	−0.45	−0.35	0.66	−0.35	1.00

#—index/number; ADC—Apparent diffusion coefficient, *T*_2_—Transversal NMR relaxation time, HU—Hounsfield units, avg—Sample mean value, var—Within sample variability (standard deviation), RBC %—Percentage of red blood cells in the thrombus, /—None, AA—Anti-aggregation, AC—Anticoagulant, NIHSS—NIH stroke scale, mRS—Modified Rankin scale, i—Initial, Δ—Difference between final and initial.

## Data Availability

The data presented in this study are available on request from the corresponding author.
